# Supplementary material for: Physiological and Biochemical Characteristics of Rainbow Trout with Severe, Moderate and Asymptomatic Course of Vibrio anguillarum Infection
Source: Animals (Basel). 2022 Oct 1;12(19):2642. doi: 10.3390/ani12192642 (PMC9559680; doi:10.3390/ani12192642)
Supplement: Supplementary file 1 [file animals-12-02642-s001.zip › SupplementaryFigures S1-S3.pdf]

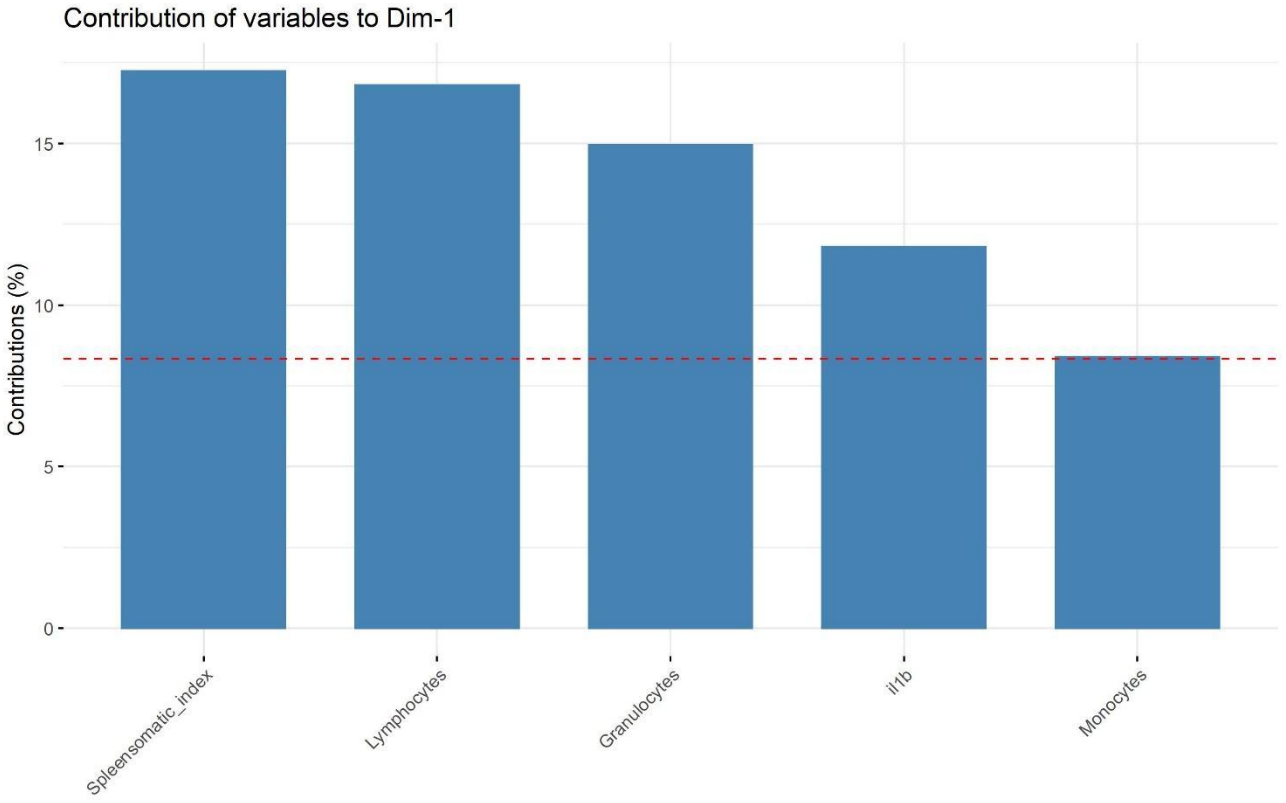

Figure S1. Contribution of variables to dimension 1 in the principal component analysis of the measured morphophysiological parameters and biomarkers of immune response of rainbow trout collected in the fish farm during a vibriosis outbreak.

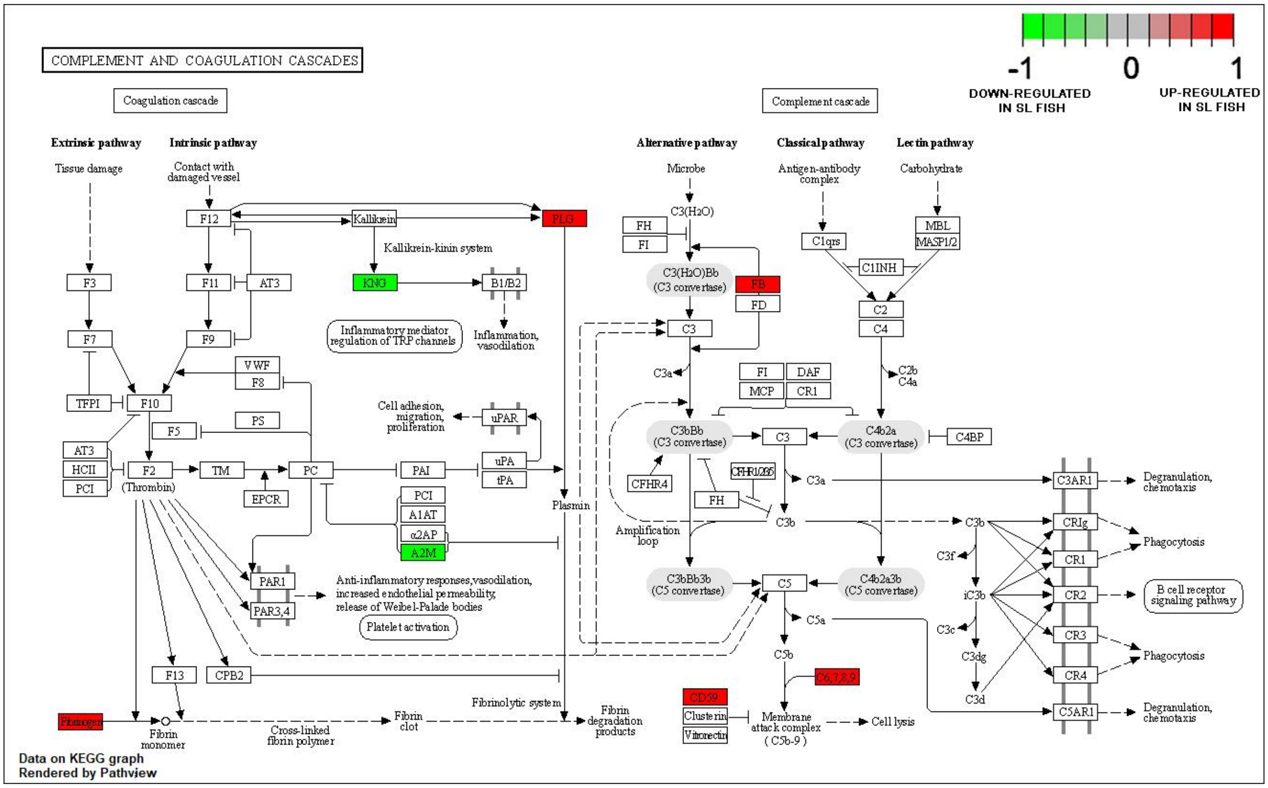

Figure S2. Reconstruction of the complement and coagulation cascade KEGG pathway (ko04610) in severely affected (SL) trout compared to other fish in the fish farm during a vibriosis outbreak.

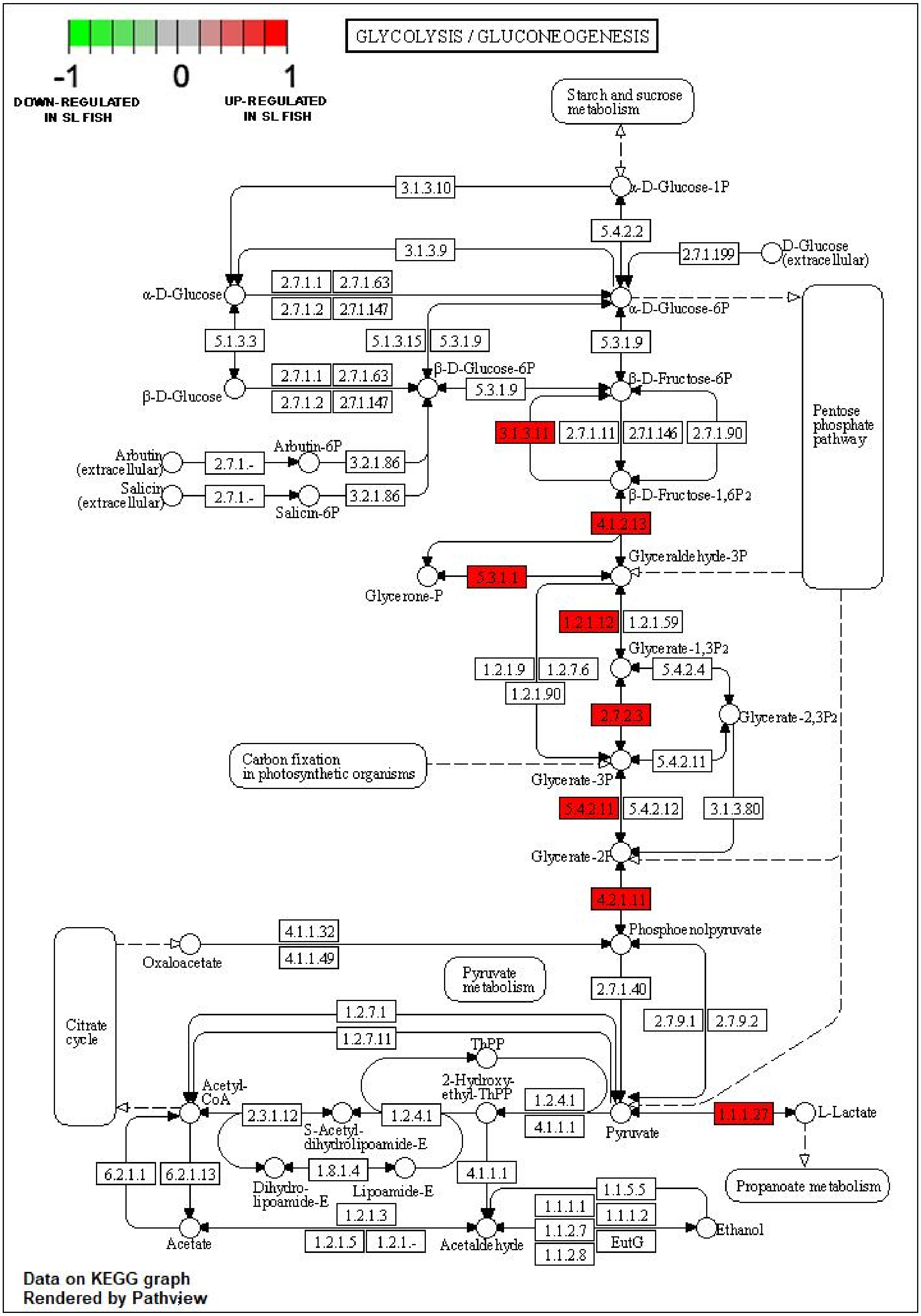

Figure S3. Reconstruction of the glycolysis/gluconeogenesis KEGG pathway (ko00010) in severely affected (SL) trout compared to other fish in the fish farm during a vibriosis outbreak.
